# Physiological and morphological responses of *Pinus ponderosa* seedlings to moisture limitations in the nursery and their implications for restoration

**DOI:** 10.3389/fpls.2023.1127656

**Published:** 2023-05-10

**Authors:** Jeremiah R. Pinto, Joshua L. Sloan, Gokhan Ervan, Owen T. Burney

**Affiliations:** ^1^ Rocky Mountain Research Station, United States Forest Service, Moscow, ID, United States; ^2^ Department of Forestry, New Mexico Highlands University, Las Vegas, NM, United States; ^3^ John T. Harrington Forestry Research Center, New Mexico State University, Mora, NM, United States

**Keywords:** drought conditioning, seedling propagation, drought stress, hydraulically active xylem, nursery irrigation

## Abstract

Successful establishment of *Pinus ponderosa* seedlings in the southwestern United States is often limited by stressful and harsh site conditions related to drought severity and severe disturbances such as wildfire and mining operations. Seedling quality has an important influence on outplanting performance, but nursery practices that typically employ optimal growing environments may also be limiting seedling morphological and physiological performance on stressful outplanting sites. To address this, a study was established to test alterations in seedling characteristics subjected to irrigation limitations during nursery culture and their subsequent outplanting performance. This study was conducted as two separate experiments: (1) a nursery conditioning experiment examined seedling development of three New Mexico seed sources exposed to three irrigation levels (low, moderate, and high); (2) a simulated outplanting experiment examined a subset of the seedlings from experiment 1 in a controlled outplanting environment consisting of two soil moisture conditions (mesic, maintained via irrigation and dry, irrigated only once). In the nursery study, the lack of interactions between seed source and irrigation main effects for most response variables indicate that low irrigation treatment level responses were consistent across a range of sources. Irrigation treatment levels from the nursery resulted in few morphological differences; however, the low irrigation level increased physiological parameters such as net photosynthetic rate and water use efficiency. In the simulated outplanting experiment, seedlings subjected to less irrigation in the nursery had greater mean height, diameter, needle dry mass, and stem dry mass; additionally, low irrigation levels in the nursery increased the amount of hydraulically active xylem and xylem flow velocity. Overall, this study shows that nursery culture irrigation limitations, regardless of the seed sources tested, can improve seedling morphology and physiological functioning under simulated dry outplanting conditions. This may ultimately translate to increased survival and growth performance on harsh outplanting sites.

## Introduction

Forest restoration planting efforts on degraded landscapes, including post-fire and mine reclamation sites, are often hindered by abiotic and biotic stress factors that influence seedling performance (Grossnickle, 2005; Anjum et al., 2011; Grossnickle, 2012). Of these stress factors, water availability is often the most limiting factor and results in plant moisture stress levels that adversely affect plant physiology and morphology (Galmés et al., 2007; Claeys and Inzé, 2013) and increase the likelihood of seedling mortality (Allen et al., 2010; Flathers et al., 2016; Mildrexler et al., 2016). Making forest restoration efforts more challenging is the added factor of climate change, which is predicted to increase the frequency and severity of drought conditions, especially in the southwestern United States (Allen et al., 2010; Allen et al., 2015).

*Pinus ponderosa* (Lawson and C. Lawson var. *scopulorum* Engelmann) is a drought-tolerant conifer species (Vance and Zaerr, 1991) used for reforestation and restoration in the western United States (Oliver and Ryker, 1990; Ouzts et al., 2015). However, the survival rate of planted *P. ponderosa* seedlings in the southwestern region of the US is approximately 25% (Ouzts et al., 2015), though this estimate does not address survival rate differences between nursery stock types, time of planting, animal browsing pressure, and site preparation activities (e.g., vegetation control). Evaluations of success that do not take these factors into account limit our ability to identify cultural practices in the nursery and in the field that should be optimized to increase survival rates (Grossnickle, 2012).

The Target Plant Concept (TPC; Landis, 2011; Dumroese et al., 2016; Davis and Pinto, 2021) highlights the need to define seedling traits (morphological and physiological) that improve seedling performance for specific outplanting environments. Past studies have indicated that target morphological traits for drought conditions might include seedlings with larger or longer root systems (Chirino et al., 2008; Pinto et al., 2012) or seedlings with balanced root-to-shoot ratios (Cregg, 1994). Target physiological and morphological characteristics also point towards nutrient-rich and larger seedlings for dry outplanting sites (Oliet et al., 2009). Many restoration programs and forest nurseries do not consider the TPC, resulting in poor outplanting performance (Haase and Davis, 2017). Incorporating the TPC into all phases of restoration planting efforts results in a higher likelihood of success (Dumroese et al., 2016).

Development of improved nursery cultural practices can lead to increased outplanting success. Historically and currently, nursery practices have aimed to provide optimal growing conditions from the time seeds are sown until lifting for either outplanting or storage (van den Driessche, 1991a; Haase and Davis, 2017). Optimal nursery growing environments are designed to control root media moisture via irrigation, nutrients via fertilization, temperature levels via heaters and coolers, and other critical environmental factors (Landis et al., 1990). However, these optimal conditions may produce physiological and morphological plant trait characteristics that do not match those required for outplanting success on harsh sites, resulting in failure to restore these sites (Grossnickle, 2012).

Soil moisture represents one of the most limiting factors on harsh outplanting sites (Holden et al., 2015). Conditioning seedlings for low soil moisture outplanting conditions by manipulating irrigation during nursery culture could be an effective strategy to improve outplanting success (Sloan et al., 2020). This strategy would be especially useful if conditioning treatments were effective across a range of seed sources (illustrating a potentially manipulative plastic phenotypic trait) and would prove to be a useful cultural tool for nursery managers. Seedlings exposed to such drought conditioning treatments before outplanting have shown tolerance to water stress with improved water status and gas exchange, as well as enhanced survival rates in the field (van den Driessche, 1991a; van den Driessche, 1991b; Abidine et al., 1994). Conditioned seedlings were also observed to continue photosynthesizing at lower soil moisture levels than seedlings never exposed to conditioning (Ashton, 1956; Ackerson and Hebert, 1981; Sloan et al., 2020). Additionally, *P. ponderosa* seedlings exposed to drought stress during the greenhouse growth period, versus seedlings that were well-watered, lived longer in the field (Cregg, 1994).

Nursery cultural practices influencing xylem structure and function may benefit restoration objectives by decreasing drought-induced mortality. Even though water moves through xylem passively, the structure and utilization of these xylem elements can influence conductivity and resistance to drought stress (Venturas et al., 2017). When plant mortality occurs via drought, it is often a result of hydraulic failure (e.g., cavitation) linked to xylem structure and function (McDowell et al., 2013; Anderegg and Hille Ris Lambers, 2016; Rodríguez-Calcerrada et al., 2017). Therefore, changes made in xylem structure and function during the nursery growth phase may help to provide a buffer against hydraulic failure under water-limited conditions at the outplanting site. Additionally, changes to the amount of hydraulically active xylem, as a percentage, may also provide a buffer to improve overall plant performance and reduce mortality as it relates to plant moisture stress (Jacobsen et al., 2015).

The objective of this study was to examine the effects of reduced water delivery during the nursery growth phase on the structure and function of *P. ponderosa* with the aim of improving survival and growth on dry, harsh outplanting sites. Additionally, this study assessed, to a limited extent, the potential interactions between seed source (i.e., genetic influence) and water limitations during nursery production (i.e., environmental influence). To do this, two experiments were conducted: a nursery conditioning experiment and a simulated outplanting experiment. We hypothesized that: 1) drought-conditioned seedlings would exhibit morphological and physiological traits that are beneficial for dry outplanting sites (e.g. more roots, balanced root-to-shoot ratio, greater percentage of hydraulically active xylem); and 2) there would be no interaction between seed source and drought conditioning treatments.

## Methods

### Plant material

Both experiments were conducted at New Mexico State University’s John T. Harrington Forestry Research Center greenhouse (35° 97’ 63” N, 105° 34’ 81” W) in Mora, NM. For both the nursery conditioning experiment and the simulated outplanting experiment (hereafter NCE and SOE, respectively), three *P. ponderosa* seed sources were selected based on variations in mean annual precipitation and mean annual temperature conditions that significantly affect ponderosa pine growth in the southwestern US (Adams and Kolb, 2005; Puhlick et al., 2021). The Valle Vidal Canyon source was collected from the Carson National Forest (36° 45’ 07” N, 105° 16’ 09” W). The Manzano Canyon source was located 40 km southeast of Albuquerque, NM (34° 36’ 12” N, 106° 21’ 39” W). The Philadelphia Canyon source was located 16 km north of Ruidoso, NM (33° 27’ 05” N, 105° 41’ 39” W). Climate data for each location are in **Table 1**.

**Table 1:** Seed source locations (New Mexico, USA), elevation (above sea level), and annual climate metrics.

Seed Source Location	Elevation (m a.s.l.)	PPT (mm)	T_min_ (C)	T_mean_ (C)	T_max_ (C)	VPD_max_ (kPA)
Valle Vidal	2950	591	-11.2 (Jan)	4.5	22.5 (Jul)	1.95 (Jul)
Manzano Canyon	2239	524	-7.5 (Jan)	9.4	28.8 (Jul)	3.14 (Jun)
Philadelphia Canyon	2319	672	-4.9 (Jan)	9.5	25.7 (Jun)	2.59 (Jun)

PPT is precipitation; T_min_, T_mean_, and T_max_ are minimum, average, and maximum temperatures, respectively; and VPD_max_ is maximum vapor pressure deficit. Months in parentheses are the times in which each metric occurs.

Seeds from all three sources were sown on 15 June 2015, in 164 mL containers (SC10 Super, Ray Leach Containers, Stuewe and Sons, Inc., Tangent, OR, USA) containing a 2:1:1 (v:v:v) mixture of sphagnum peat, perlite, and vermiculite. Containers were grouped into racks with 98 seedlings per rack. After sowing, racks were subjected to regular overhead misting irrigation with clear water (pH 6.0) five times per day until germination. Following germination and prior to the initiation of irrigation treatments, racks were irrigated to 100% of container water holding capacity upon drying down to a target of 85% of container water holding capacity, determined gravimetrically (Dumroese et al., 2015). Greenhouse conditions were maintained at 29.4/18.3°C (day/night), and natural light was supplemented as needed to maintain an 18-hour photoperiod.

### Experiment 1: Nursery conditioning experiment (NCE)

The NCE utilized a randomized complete block (RCB) design using a 3 × 3 factorial treatment structure based on three seed sources and three irrigation levels (High, Moderate, and Low). Treatment combinations were replicated over four blocks where racks were randomly assigned to blocks and treatments. Individual treatment combinations were applied to a single rack (i.e., experimental unit) of 98 seedlings within each block. Methods for applying irrigation treatments and fertilization were nearly identical to those used by Sloan et al. (2020). The only difference was in the target dry-down values of the irrigation levels. The three irrigation levels were based on gravimetric percent moisture content of containers using the “Manager’s Technique” (whole system: media, seed covering, tray, container, and water) described in Dumroese et al. (2015) in which irrigation targets were High - 85%, Moderate - 70%, and Low - 55%. Translating these percentages into the “Scientific Technique” (water only) from Dumroese et al. (2015), values were calculated as High - 76%, Moderate - 52%, and Low - 28%. Irrigation treatments and fertilization were initiated on 13 July 2015, and ran until the destructive sampling was conducted during the first week of October 2015.

All seedlings were irrigated to full saturation the day before sampling. At the time of destructive sampling, five seedlings were randomly sub-sampled from each experimental unit (i.e., rack) for measurements. All experimental units for a single block were measured in a single day. Total seedling height (measured from the root collar to the base of the terminal bud) and root collar diameter (RCD) were measured for all five sub-sampled seedlings. Three of the five measured seedlings were randomly selected for further measurements of photosynthesis, root volume, first order lateral roots, and biomass (108 total seedlings). Additionally, two of these three seedlings were randomly selected for measurements of plant hydraulic properties and needle characteristics.

Net photosynthetic rate and water use efficiency (calculated as net photosynthesis/stomatal conductance) were measured on the three subsampled seedlings per treatment combination per block. A mid-canopy fascicle from the southern side of each seedling was selected for gas exchange measurements. All three needles from the fascicle were taped together at the ends, and the central un-taped length of needles was placed into the leaf chamber of a portable, open-system, infrared gas analyzer (LI-6400XT Portable Photosynthesis System) equipped with a red/blue LED light source (6400-02B LED, LI-COR, Inc., Lincoln, NE, USA). Measurements were taken between 11:00 and 15:00 with instrument settings as follows: block temperature = 20°C, CO_2_ reference = 375 µmol mol^-1^, flow rate = 500 µmol s^-1^, and photosynthetically active photon flux density in the leaf chamber (PAR_i_) = 1500 µmol m^-2^ s^-1^. Leaf (i.e., needle) areas used in net photosynthetic rate and water use efficiency calculations were based on methods found in Pinto et al. (2012).

First order lateral roots (FOLR) and root volume measurements were made for the three sub-sample seedlings. Seedlings were removed from their containers and the media was washed from the roots. The root volume was measured gravimetrically using the water displacement method (Burdett, 1979), and FOLR greater than 1 cm in length were counted.

Two of the three seedlings selected for destructive harvest were randomly selected for hydraulically active xylem, xylem flow velocity, total needle area, and stomatal density measurements. For measurements of hydraulically active xylem and xylem flow velocity, a xylem staining method was used based on Jacobsen et al. (2015). Stems of selected seedlings were severed underwater at the root collar using a razor blade. The intact aboveground portion of the seedling was quickly submerged 1 cm into a 4.5 mL cuvette containing 4.0 mL of a 0.01% (w:v) crystal violet: deionized water solution at a pH of 2.0. Environmental conditions during staining included a metal halide light source that provided 540 µmol m^-2^ s^-1^ PPFD and an ambient temperature of 29.4°C. Seedlings were maintained in the dye solution for 20 min. Upon removal from a cuvette, a razor blade was used to sever the base of the stem 2 cm above the root collar, from which point a 0.1 mm thick cross-section was excised for microscopic analysis of xylem properties. A light microscope with an attached digital camera captured images using a 4X objective lens. Images captured from the microscope were analyzed using graphics software (Adobe Photoshop CC 2017, Adobe Systems Inc., San Jose, CA, USA) to measure the percentage of total xylem area stained by the crystal violet solution.

After sub-sampled seedlings were removed from the staining process to measure hydraulically active xylem, a razor blade was used to sever each stem at 1 cm increments, moving upward from the root collar. Xylem flow velocity was determined by visually identifying (with the aid of a hand lens) the upper limit of dye occurrence within each seedling. Xylem flow velocity, in cm h^-1^, was then calculated as follows: xylem flow velocity in cm h^-1^ = (stem distance traveled by dye in cm over 20 min) × 3.

One randomly selected subsampled seedling was slated to measure native stem and leaf specific conductance. Stem segments 1.5–2.5 cm in length were severed just above the root collar underwater with a razor blade. Bark was removed, and both ends were freshly trimmed (<1 mm) underwater. Conductance measurements followed the procedure of Kavanagh and Zaerr (1999), connecting stems to solution-filled tubing with a reservoir placed 0.5 m above the samples. The solution was filtered, deionized, and degassed. A 4.5-mL cuvette (pre-weighed) with cotton gauze was used to capture stem conductance solution. The vial was measured at periodic 120-s time intervals. Stem conductance (*K*_s_) was calculated by dividing the flow rate across the segment by the pressure gradient and stem cross-sectional area. Leaf-specific (*K*_l_) conductance was calculated similarly but with the addition of needle area (needle area measurements described below).

To measure needle area, all needles were removed from the two seedlings that had been subsampled for xylem measurements. All needles from each sub-sampled seedling were arranged on a flatbed scanner, and high-resolution images were captured. Total needle area was determined from the stored images using graphics software (Adobe Photoshop CC, Adobe Systems Inc., San Jose, CA, USA). To obtain adaxial stomatal density, the middle section of the first mature needle from the bottom of each seedling was placed under a light microscope (AmScope, MU1400; 4x objective lens) for digital image capture. Using the graphics software, a grid was created to measure the stomata within a 1 mm^2^ section of the needle.

After the completion of all other measurements, dry masses were measured for needles, stems, and roots of each of the three sub-samples after drying to constant mass at 68°C for 48 h. Dry masses were recorded for each organ of each subsample. Total seedling dry mass was calculated as the sum of the individual organ mass for each sub-sampled seedling, and root:shoot ratio was calculated by dividing the root dry mass of each sample by the sum of the stem and needle dry masses for that seedling. After dry mass acquisition, all tissue samples were ground (Wiley mill, Thomas Scientific, Swedesboro, NJ, USA) to pass through a 20-mesh screen in preparation for analysis of carbon isotope ratios.

Aliquots of ground needle tissue from the three sub-samples were submitted to the Stable Isotope Core Laboratory of Washington State University for determination of δ^13^C. Samples for carbon are converted to CO_2_ with an elemental analyzer (ECS 4010, Costech Analytical, Valencia, CA) and then separated with a 3-m gas chromatograph column and analyzed with a continuous flow isotope ratio mass spectrometer (Delta PlusXP, Thermofinnigan, Bremen). Isotope reference materials are interspersed with samples for calibration. Carbon isotope ratios were expressed relative to the Pee Dee River belemnite standard (Craig, 1957).

Post sampling, new saturation weights were acquired and seedlings were moved to an outside shade structure for hardening. All treatments and seedlings were irrigated at 65% gravimetric moisture content (Manager’s technique; Dumroese et al., 2015) until they were moved into cold storage. In December, all seedlings were moved into cold storage and maintained at an average temperature of 2.5 C. Seedlings were held in cold storage until 1 June 2016. Upon removal, they were fully saturated prior to being used for the second experiment.

### Experiment 2: Simulated outplanting experiment (SOE)

The SOE utilized a RCB design with a 3 × 3 × 2 factorial treatment structure derived from the addition of a post-transplant simulated drought factor (two levels: Drought vs. Control) to the original 3 × 3 factorial treatment structure of the NCE. Four seedlings were randomly selected from each treatment by block combination (i.e., each rack) from the NCE seedlings held over in cold storage for a total of 144 seedlings. Each seedling constituted an experimental unit, and the blocking structure established during the NCE was maintained throughout the SOE.

On 7 June 2016, the 144 seedlings selected for inclusion in the SOE were transplanted into 25.4 cm tall x 10.2 cm diameter pots (Treepots™, Stuewe and Sons, Inc., Tangent, OR, USA), with a volume of 2.79 L and filled with a 1:1 mixture by volume of sand and perlite. Seedlings did not receive any additional fertilizer during the SOE. At the time of transplant, the four seedlings within each NCE treatment by block combination were randomly assigned to one of two simulated drought levels: a post-transplant Drought level (two seedlings) or a post-transplant well-watered Control level (two seedlings). Each seedling in the Drought level was randomly paired with a seedling from the Control level and randomly assigned to be harvested for destructive sampling at one of two harvest times: 1) based on the “Scientific Technique” when the water mass of an individual container dropped to 55% of the total water mass at container capacity (Initial Harvest), or 2) when a seedling assigned to the Drought level exhibited 100% needle mortality (Final Harvest). All containers were irrigated to saturation following transplanting. Control seedlings were watered to saturation whenever the water mass of an individual container dropped to 70% of the total water mass at container capacity determined by the “Scientific Technique.” The Drought level seedlings received no further irrigation throughout the experiment. The paired seedling in the Control level was harvested when its corresponding seedling in the Drought level reached its assigned dry-down condition. Greenhouse environmental and photoperiod conditions during the SOE were maintained to be similar to those of the NCE.

For the Initial Harvest, height, RCD, hydraulically active xylem, xylem flow velocity, needle area, stomatal density (both adaxial and abaxial), root dry mass, stem dry mass, needle dry mass, FOLR, and root volume were measured using the same protocols as described for the NCE. However, during image analysis, the xylem area was separated into Year-1 and Year-2 xylem areas. Each of the xylem years was analyzed separately for the percentage of hydraulically active xylem. On average, the initial harvest was 57 days (range: 50-68 days) post-transplant. For the Final Harvest, only dry mass by organ was measured. The final harvest averaged 128 days (range: 113-161 days) post-transplant.

### Statistical analyses

In the NCE, the effects of seed source, irrigation level, and the interaction of these two factors on morphological and physiological responses were analyzed using analysis of variance (ANOVA). In the SOE, the effects of seed source, irrigation level, drought level, and all associated interactions of these factors on morphological and physiological responses were analyzed using ANOVA. For both experiments, all data were analyzed using the PROC MIXED procedure in SAS (SAS Institute Inc., Cary, NC, USA) with α = 0.05. Tukey’s Honest Significant Difference test was used to detect significant differences between means (α = 0.05). When interactions were found to be non-significant, lower-order terms were reported. All residuals were checked for constant variance and normality.

## Results

### Nursery conditioning experiment

No interactions were observed between seed source and irrigation treatments for seedling height, root collar diameter, root-to-shoot ratio, total dry mass, root dry mass, stem dry mass, needle dry mass, root volume, first-order lateral roots, or water use efficiency (*p* > 0.05). Seed source was not found to influence root-to-shoot ratio (*p* = 0.9762), root volume (*p* = 0.1748), or water use efficiency (*p* = 0.5629), although it did influence seedling height (*p* < 0.0001), root collar diameter (*p* < 0.0001), the number of first-order lateral roots (*p* = 0.0317), root dry mass (*p* = 0.0059), stem dry mass (*p* < 0.0001), needle dry mass (*p* = 0.0160), and total dry mass (*p* = 0.0026). For total dry mass, root dry mass, stem dry mass, height, and diameter, the Manzano and Philadelphia Canyon seed sources were larger across parameters compared to the Valle Vidal seed source (**Table 2**). For needle dry mass and the number of first-order lateral roots, the Manzano seed source was larger and had more roots than the Valle Vidal seed source, but the Philadelphia Canyon seed source did not differ from either of the other sources.

**Table 2:** Seedling morphological and physiological characteristics by seed source for the Nursery Conditioning Experiment.

Nursery Conditioning Experiment Parameter	Seed Source		
	Valle Vidal	Manzano Canyon	Philadelphia Canyon
Total seedling dry mass (g)	0.95 (0.03) b	1.18 (0.06) a	1.13 (0.05) a
Needle dry mass (g)	0.55 (0.03) b	0.66 (0.03) a	0.61 (0.03) ab
Stem dry mass (g)	0.10 (0.01) b	0.14 (0.01) a	0.15 (0.01) a
Root dry mass (g)	0.30 (0.01) b	0.38 (0.02) a	0.37 (0.02) a
Total height (cm)	5.3 (0.2) b	8.6 (0.2) a	8.5 (0.2) a
Root collar diameter (mm)	2.3 (0.0) b	2.5 (0.0) a	2.6 (0.0) a
FOLR (#)	34.5 (1.2) b	38.5 (1.1) a	38.0 (1.3) ab
Root volume (cm^3)^	3.11 (0.17) a	3.22 (0.16) a	4.27 (0.80) a
Root : Shoot (g:g)	0.48 (0.02) a	0.48 (0.02) a	0.48 (0.01) a
WUE (µmol CO_2_ mmol H_2_O^-1^)	71.9 (3.2) a	75.3 (4.1) a	72.3 (4.1) a
A_net_ (µmol m^-2^ s^-1^)	10.05 (0.38) a	11.14 (0.48) a	10.51 (0.38) a
Total needle area (cm^2^ seedling^-1^)	45.7 (1.7) b	55.1 (1.9) a	54.8 (1.8) a
Stomatal density (abaxial; # mm^-2^)	54.7 (2.89) a	53.4 (1.58) a	54.8 (2.87) a
% Active xylem	49.8 (5.01) a	50.8 (6.81) a	41.4 (7.46) a
Xylem flow velocity (cm h^-1^)	7.5 (0.32) a	9.9 (1.00) a	8.1 (0.79) a
δ^13^C (‰)	-28.91 (0.09) a	-29.07 (0.10) a	-29.02 (0.11) a

Values displayed are the mean (± standard error of the mean) of total seedling dry mass, needle dry mass, stem dry mass, root dry mass, height, root collar diameter, number of first order lateral roots (FOLR), root volume, root-to-shoot ratio, water use efficiency (WUE), net photosynthetic rate (A_net_), total needle area, abaxial stomatal density, percent active xylem, xylem flow velocity, and stable carbon isotopes (δ^13^C). Within each parameter, means followed by the same letter do not differ significantly (α = 0.05).

Irrigation treatments were not found to influence root dry mass, stem dry mass, needle dry mass, total dry mass, or root volume (*p* > 0.05), but did influence seedling height (*p* < 0.0001), diameter (*p* = 0.0147), the number of first order lateral roots (*p* = 0.0226), root-to-shoot ratio (*p* = 0.0027), and water use efficiency (*p* = 0.0002). The Moderate irrigation level resulted in larger seedlings, in both height and diameter, relative to the High and Low treatments (**Table 3**). The Low treatment resulted in fewer first-order later roots relative to the High and Moderate treatments. The High treatment yielded a larger root-to-shoot ratio than the other two irrigation treatments.

**Table 3:** Seedling morphological and physiological characteristics by irrigation level for the Nursery Conditioning Experiment.

Nursery Conditioning Experiment Parameter	Irrigation Level		
	High	Moderate	Low
Total seedling dry mass (g)	1.09 (0.05) a	1.11 (0.07) a	1.05 (0.04) a
Needle dry mass (g)	0.59 (0.02) a	0.62 (0.04) a	0.60 (0.03) a
Stem dry mass (g)	0.13 (0.01) a	0.14 (0.01) a	0.12 (0.01) a
Root dry mass (g)	0.37 (0.02) a	0.35 (0.03) a	0.33 (0.01) a
Total height (cm)	7.8 (0.3) b	8.0 (0.3) a	6.6 (0.2) b
Root collar diameter (mm)	2.4 (0.0) b	2.6 (0.1) a	2.4 (0.0) b
FOLR (#)	37.7 (1.2) a	38.9 (1.3) a	34.5 (1.1) b
Root volume (cm^3^)	3.46 (0.12) a	4.21 (0.81) a	2.93 (0.18) a
Root : Shoot (g:g)	0.52 (0.01) a	0.46 (0.01) b	0.46 (0.01) b
WUE (µmol CO_2_ mmol H_2_O^-1^)	65.0 (3.2) b	73.2 (5.0) ab	81.3 (2.3) a
A_net_ (µmol m^-2^ s^-1^)	9.6 (0.3) b	10.7 (0.41) ab	11.5 (0.39) a
Total needle area (cm^2^ seedling^-1^)	51.2 (1.8) ab	55.0 (1.9) a	49.4 (2.02) b
Stomatal density (abaxial; # mm^-2^)	55.6 (2.89) a	57.4 (1.49) a	49.9 (2.42) a
% Active xylem	45.0 (5.88) a	50.8 (8.36) a	46.3 (5.07) a
Xylem flow velocity (cm h^-1^)	7.8 (0.52) a	9.0 (1.1) a	8.8 (0.71) a
δ^13^C (‰)	-29.33 (0.07) a	-29.21 (0.08) a	-28.45 (0.07) b

Values displayed are the mean (± standard error of the mean) of total seedling dry mass, needle dry mass, stem dry mass, root dry mass, height, root collar diameter, number of first order lateral roots (FOLR), root volume, root-to-shoot ratio, water use efficiency (WUE), net photosynthetic rate (A_net_), total needle area, abaxial stomatal density, percent active xylem, xylem flow velocity, and stable carbon isotopes (δ^13^C). Irrigation levels were High, Moderate, and Low (85, 70, and 55% of container water-holding capacity, respectively). Within each parameter, means followed by the same letter do not differ significantly (α = 0.05).

No interactions between seed source and irrigation treatments were observed for net photosynthetic rates, needle area, or abaxial stomatal density (*p* > 0.05). An interaction was observed, however, for adaxial stomatal density (*p* = 0.0162), in which seedlings from the Valle Vidal seed source grown under the High irrigation level had fewer adaxial stomata when compared to seedlings from the Philadelphia Canyon source grown under identical irrigation conditions (data not shown).

**Figure 1:**
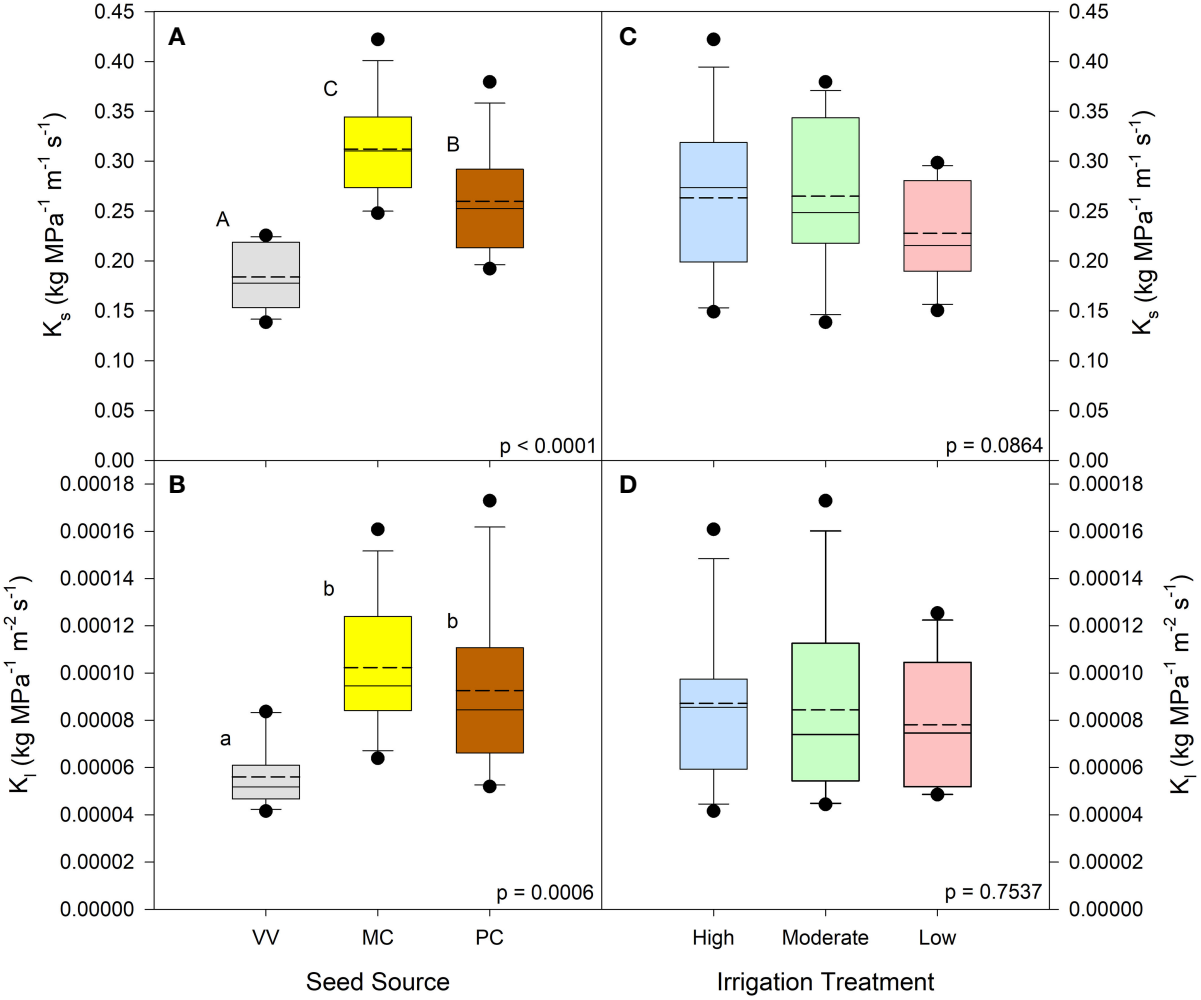
*Pinus ponderosa* seedling stem conductance by seed source and irrigation treatment. Native stem conductance (K_s_) is shown in panels **(A, C)**; leaf specific stem conductance (K_l_) is shown in panels **(B, D)**. Dashed lines are mean conductance values; solid lines are medians. Seed source abbreviations: Valle Vidal (VV), Manzano Canyon (MC), and Philadelphia Canyon (PC); all seed sources are located in New Mexico, USA. Irrigation treatment abbreviations are: High, Medium, and Low (85, 70, and 55% of container water-holding capacity, respectively). Within each parameter, means followed by the same letter do not differ significantly (α = 0.05).

Seed source was not found to influence net photosynthetic rates (*p* = 0.0984) or abaxial stomatal density (*p* = 0.8904). It was shown, however, to influence needle area (*p* < 0.0001), where seedlings from the Valle Vidal seed source had the smallest needle area (**Table 2**). The irrigation treatment likewise did not influence abaxial stomatal density (*p* = 0.0869) but did influence net photosynthetic rates (*p* = 0.0025) and needle area (*p* = 0.0373) (**Table 3**). The Low irrigation level led to an increased net photosynthetic rate relative to the High level and reduced needle area relative to the Moderate level. With regards to seed source or irrigation treatments, no significant interactions or main effects were observed for xylem flow velocity or active xylem area (*p* > 0.05; **Tables 2**, **3**). Seed source did yield a significant response for both *K*_s_ and *K*_l_ (p ≤ 0.0006). *K*_s_ was highest for the Manzano Canyon source and lowest for the Valle Vidal source; the Valle Vidal source elicited a lower *K*_l_ compared to the other two seed sources (**Figures 1A, B**). Nursery irrigation treatments had no effect on either *K*_s_ or *K*_l_ ( p ≥ 0.0864; **Figures 1C, D**).

Mean carbon isotope ratios (δ ^13^C) for needle samples were found to differ significantly only for the irrigation treatment main effect (*p* > 0.0001). Results indicate the Low irrigation treatment level had enriched levels of δ ^13^C compared to the other two irrigation levels (**Table 3**).

### Simulated outplanting experiment – initial harvest

No three-way interactions were observed for any initial harvest response variables (*p* > 0.05). The only significant interaction for morphological parameters was observed for root dry mass between seed source and the simulated drought factor. The Manzano Canyon seed source under the Drought level of the simulated drought factor had a larger root system, by mass, compared to all other seed sources and simulated drought treatment combinations except for the Philadelphia Canyon seed source under the Drought level (*p* = 0.0105) (data not shown).

Seed source did not influence the number of first-order lateral roots, the root-to-shoot ratio, or needle dry mass. Still, it did influence seedling height (*p* < 0.0001), diameter (*p* < 0.0001), root volume (*p* = 0.0493), stem dry mass (*p* < 0.0001), and total dry mass (*p* = 0.0053).

At the initial SOE harvest, NCE irrigation treatments had no detectable influence on the number of first-order lateral roots or the root-to-shoot ratio (*p* > 0.05). It did influence, however, seedling height (*p* < 0.0001), root collar diameter (*p* < 0.0001), root volume (*p* < 0.0001), root dry mass (*p* < 0.0001), stem dry mass (*p* < 0.0001), needle dry mass (*p* < 0.0001), and total dry mass (*p* < 0.0001) (**Table 4**). The Moderate and Low irrigation levels resulted in larger seedlings relative to the High irrigation level for all the above seedling parameters except seedling height. Seedling height in the Moderate irrigation level did not differ from the Low level but was greater than the High level (**Table 4**).

**Table 4:** Seedling morphological and physiological characteristics by nursery irrigation level for the Initial Harvest of the Simulated Outplanting Experiment.

Simulated Outplanting Experiment Parameter – Initial Harvest	Nursery Irrigation Level		
	High	Moderate	Low
Total seedling dry mass (g)	3.50 (0.14) a	4.73 (0.16) b	4.97 (0.18) b
Needle dry mass (g)	1.56 (0.06) a	2.17 (0.06) b	2.36 (0.07) b
Stem dry mass (g)	0.63 (0.03) a	0.85 (0.04) b	0.84 (0.05) b
Root dry mass (g)	1.31 (0.08) a	1.7 (0.09) b	1.78 (0.09) b
Total height (cm)	15.77 (0.68) a	17.84 (0.69) b	17.25 (0.76) ab
Root collar diameter (mm)	3.63 (0.08) a	4.26 (0.12) b	4.40 (0.13) b
FOLR (#)	19.79 (0.76) a	21.42 (1.20) a	20.46 (0.97) a
Root volume (cm^3^)	7.61 (0.37) a	9.42 (0.43) b	10.60 (0.44) b
Root : Shoot (g:g)	0.60 (0.03) a	0.56 (0.02) a	0.56 (0.02) a
Total needle area (cm^2^ seedling^-1^)	98.5 (4.1) a	130.34 (4.5) b	144.37 (5.3) b
Stomatal density (abaxial; # mm^-2^)	66.1 (2.27) a	73.5 (1.88) a	69.3 (3.08) a
Stomatal density (adaxial; # mm^-2^)	65.0 (2.48) a	70.5 (2.33) ab	74.3 (2.53) b
% Active first year xylem	18.8 (4.54) a	33.4 (6.19) b	24.0 (5.68) ab
% Active second year xylem	11.9 (3.12) a	27.1 (4.24) b	28.0 (4.64) b
Xylem flow velocity (cm h^-1^)	3.13 (0.34) a	4.38 (0.40) a	4.13 (0.48) a

Values displayed are the mean (± standard error of the mean) of total seedling dry mass, needle dry mass, stem dry mass, root dry mass, height, root collar diameter, number of first order lateral roots (FOLR), root volume, root-to-shoot ratio, total needle area, stomatal density (abaxial and adaxial), percent active first year xylem, percent active second year xylem, xylem flow velocity. Within each parameter, means followed by the same letter do not differ significantly (α = 0.05).

No influence of simulated drought was observed for seedling root collar diameter, root volume, number of first order lateral roots, or needle dry mass (*p* > 0.05). However, differences were observed for seedling height (*p* = 0.002), root-to-shoot ratio (*p* = 0.0032), stem dry mass (*p* = 0.0059), and total dry mass (*p* = 0.0026). Seedlings exposed to the Drought treatment had greater heights, root-to-shoot ratios, stem dry mass, and total dry mass compared to the Control (data not shown).

No interactions were observed during the initial harvest for any of the needle associated parameters (*p* > 0.05). No effect of the seed source was observed for adaxial stomatal density, abaxial stomatal density, or number of needles (*p* > 0.05). No effect of irrigation treatment was observed for abaxial stomatal density or specific needle area (*p* > 0.05). No effects of simulated drought for adaxial stomatal density, number of needles, or specific needle area were observed for the Drought treatment (*p* > 0.05).

Needle area of the Philadelphia Canyon seed source was greater than in the Valle Vidal source (*p* = 0.0242) and specific needle area was lowest in the Valle Vidal source (*p* = 0.0066; data not shown). Irrigation treatments during the NCE influenced adaxial stomatal density (*p* = 0.0341), number of needles (*p* = 0.0048), and needle area (*p* < 0.0001), where decreasing water availability during the NCE tended to increase adaxial stomatal density, the number of needles, and needle area during the second year of growth (**Table 4**). Seedlings exposed to the Drought treatment had lower abaxial stomatal density (*p* = 0.0046) and higher needle area (*p* = 0.0312; data not shown).

**Figure 2:**
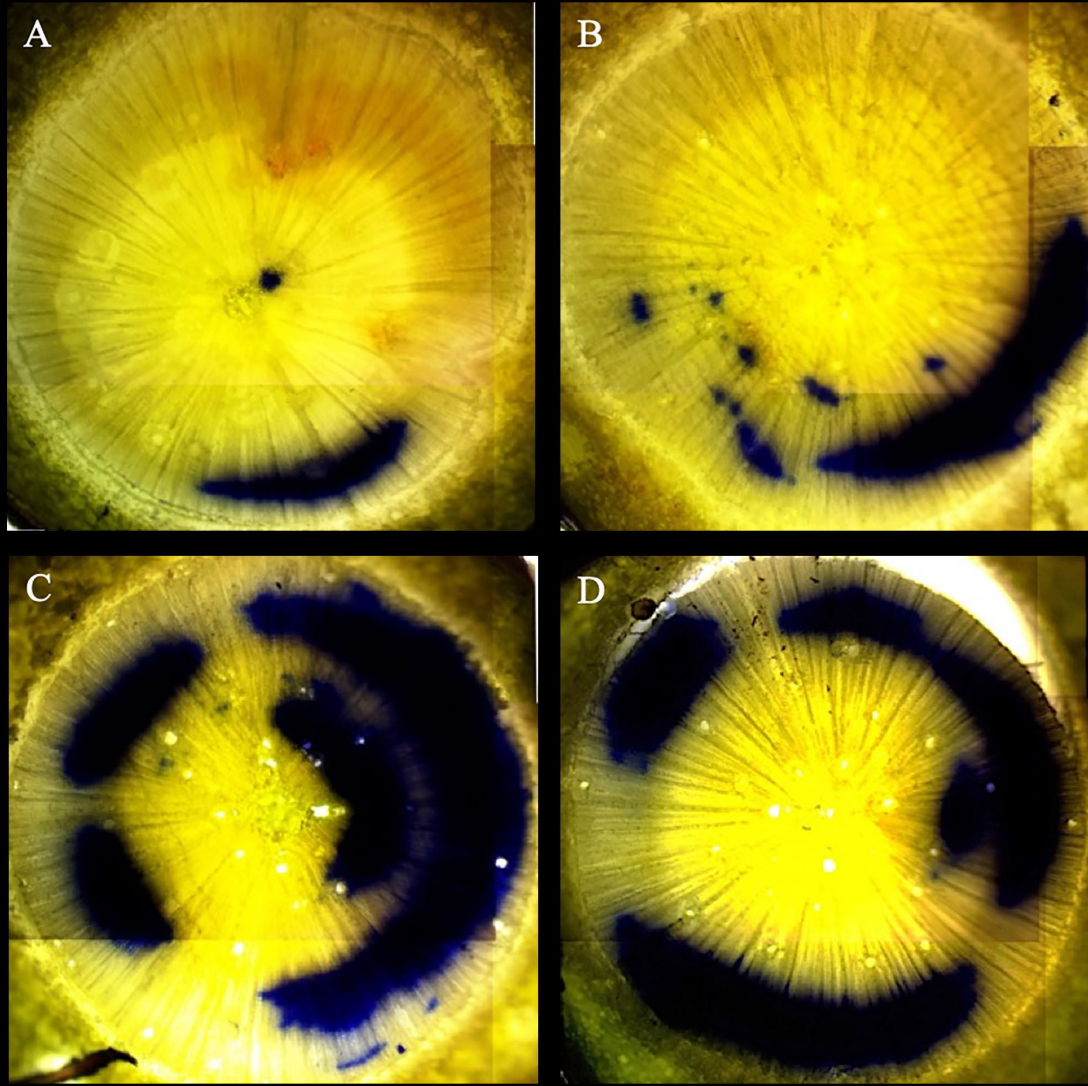
Comparison of physiologically active xylem of Initial Harvest of the Simulated Outplanting Experiment. The blue stained (crystal violet dye) areas indicate those xylem elements across two years that are actively conductive. The images displayed are representative of the High nursery irrigation level and Control simulated drought treatments **(A)**, High nursery irrigation level and Drought simulated drought treatments **(B)**, Low nursery irrigation level and Control simulated drought treatments **(C)**, and Low nursery irrigation level and Drought simulated drought treatments **(D)**. All images are from the Valle Vidal seed source.

At initial harvest, no interactions were observed for any xylem variables (*p* > 0.05). Seed source was found to influence xylem flow velocity (*p* = 0.0114), first year active xylem area (*p* = 0.0001), and second year active xylem area (*p* = 0.0014), in which the Philadelphia seed source had faster xylem flow rates and larger second year active xylem areas compared to the Valle Vidal source, and both the Manzano Canyon and Philadelphia Canyon sources had larger first year active xylem areas than the Valle Vidal source (data not shown). The irrigation treatment during the NCE did not have a detectable influence on xylem flow rates at the time of the initial harvest during the SOE (p > 0.05) but did influence first- and second-year active xylem area (*p* = 0.0485 and *p* = 0.0043, respectively; **Table 4** and **Figure 2**). Seedlings receiving the Low irrigation regime during the NCE tended to have more hydraulically active xylem than seedlings receiving the High irrigation regime (**Table 4**). Although there was no observed influence of the simulated drought factor on second year active xylem area (*p* > 0.05), seedlings in the Drought level had a faster xylem flow rate (*p* = 0.0147) and a higher first year active xylem area (*p* = 0.0212) relative to the Control level seedlings (data not shown).

### Simulated outplanting experiment – final harvest

For all morphological characteristics at final harvest, total dry mass was the only response variable with a significant interaction (simulated drought and irrigation treatment, *p* = 0.0287). Within this interaction, total dry mass for the Low irrigation level under the Control level of the simulated drought factor was greater than all other treatment combinations with the exception of the Moderate irrigation level under the Control simulated drought factor (data not shown). Stem dry mass and total dry mass were the only morphological response variables found to be influenced by seed source (*p* < 0.0001 and *p* = 0.0009, respectively). The Manzano and Philadelphia Canyon sources had higher stem and total dry mass compared to the Valle Vidal source (data not shown). Irrigation treatments during the NCE were found to influence needle, root, and stem dry mass (*p* < 0.0001, *p* = 0.0192, and *p* = 0.0001, respectively). The Low irrigation level resulted in larger seedlings relative to the High level (**Table 5**).

**Table 5:** Seedling morphological characteristics by nursery irrigation level for the Final Harvest of the Simulated Outplanting Experiment.

Simulated Outplanting Experiment Parameter – Final Harvest		Nursery Irrigation Level		
		High	Moderate	Low
Needle dry mass (g)		1.77 (0.12) a	2.50 (0.12) b	2.49 (0.17) b
Stem dry mass (g)		0.81 (0.06) a	1.07 (0.05) b	0.98 (0.06) b
Root dry mass (g)		1.80 (0.18) a	2.08 (0.16) ab	2.36 (0.23) b

Values displayed are the mean (± standard error of the mean) of needle dry mass, stem dry mass, and root dry mass. Nursery treatment irrigation levels were High, Moderate, and Low (85, 70, and 55% of container water-holding capacity, respectively). Within each parameter, means followed by the same letter do not differ significantly (α = 0.05).

## Discussion

### Nursery conditioning exriment

Nursery conditioning influenced both morphology and physiology for *Pinus ponderosa* seedlings. One of the more important findings from the NCE was the lack of interaction between seed source and irrigation treatment for most response variables. The general lack of interactions suggests that the observed responses to the irrigation treatments may be consistent across a range of seed sources for *P. ponderosa*. As a result, there is the potential for nursery managers to use these irrigation strategies in their nursery programs with similar outcomes for a range of seed sources for *P. ponderosa*. Seed source was included as a factor in this study to provide a preliminary indication of genetic variability in seedling responses to the range of irrigation treatment levels. However, in order to thoroughly test source genetic differences, genetic tests such as provenance or common garden studies should be used. These tests typically require a minimum of 50 seed sources for adequate comparison (Johnson and Lipow, 2002). Thus, interpretations of responses to the three seed sources used in this study will be difficult to decipher and are not included in this discussion with the exception of relevant interactions.

In general, the Moderate irrigation treatment yielded greater heght, root-collar diameter, and needle area morphological responses (**Table 3**). This differs from previous studies examining nursery irrigation regimes such as those with *P. ponderosa* (Olivas-García et al., 2000) and *Pinus halepensis* (Royo et al., 2001) in which the authors reported reductions in seedlings’ morphological responses (e.g., height and diameter) with decreasing soil moisture content. van den Driessche (1991a) also reported decreased root collar diameter with increased drought severity for Douglas-fir (*Pseudotsuga menziesii* (Mirb.) Franco) and white spruce (*Picea glauca* (Moench) Voss) in a nursery experiment. These studies began their irrigation treatments at much later ontological stages (104–156 weeks, 21 weeks, and 26 weeks, respectively) when seedlings had well-established secondary xylem.

In our study, irrigation treatments began at an early ontological stage (4 weeks after germination), near the onset of secondary xylem differentiation. Miller and Johnson (2017) found that *P. ponderosa* seedlings began to transition to secondary xylem between the second and third week after germination. By initiating irrigation treatments during the transition to secondary xylem development and maintaining these irrigation regimes throughout the development of the first year’s secondary xylem, it was hypothesized that whole seedling xylem characteristics and hydraulic properties could be fundamentally and lastingly altered to improve plant performance under moisture limited conditions after outplanting. Seedlings in the Moderate irrigation level resulted in higher percentages of active xylem and increased xylem flow velocities; although, these differences were not statistically significant, and followed similar patterns to morphological responses to irrigation. This lack of significant differences is likely associated with the ineffectiveness of the crystal violet dye solution for clear and high-resolution staining of the xylem structure in this species. Results from Ervan (2018) showed that crystal violet dye staining of *P. ponderosa* xylem resulted in extremely dark coloration and lateral bleeding of the dye.

Our investigation of both stem and leaf xylem conductance yielded significant effects for seed sources but not nursery irrigation. Similar results were seen by Sloan et al. (2020) in drought-conditioned aspen seedlings. Though some studies show that photosynthetic traits can scale with hydraulic conductivity in large trees (Brodribb and Field, 2000; Santiago et al., 2004), our irrigation conductance data did not align with our photosynthesis or leaf area results. Despite this, our data suggest “dry” irrigation treatments may not negatively impact hydraulic conductivity trait capacity in nursery-produced seedlings. Seed source data differences may suggest some adaptation to hot/dry climes where higher conductance levels are associated with locations that receive reduced rainfall, higher temperatures, and higher vapor pressure deficits (**Table 1**). As with photosynthetic traits, it may be more beneficial to have high capacities for gaining carbon and moving water when conditions are favorable; though, this may also be a tradeoff for vulnerability to xylem cavitation.

The High irrigation level significantly increased root volume, root:shoot ratio, and the number of first-order lateral roots in the NCE. Greater root growth and number of lateral roots were also found by Robbins and Dinneny (2018), who reported that the development of lateral roots in plants is positively related to the connection with accessible water. Such root architecture responses to water availability may provide an advantage in the uptake of both water and nutrients through the initial growth period, as observed in the NCE (Davis and Jacobs, 2005; Oliet et al., 2009). Reduction in root:shoot ratio with decreasing soil moisture has been observed with loblolly pine (*Pinus taeda* L.) and Aleppo pine (*Pinus halepensis*) by Seiler and Johnson (1988) and Villar-Salvador et al. (1999), respectively. A reduction of root biomass relative to the shoot suggests that the absorptive capacity of the root system will be less than the transpirational demands of the shoot, resulting in a higher likelihood of plant moisture stress or mortality (Walker and Huntt, 2000). In contrast, van den Driessche (1991a) reported no changes in root:shoot ratio with increasing drought severity in the nursery for Douglas-fir and white spruce because both roots and shoots of seedlings were reduced equally.

As found with root collar diameter, needle area was greatest at the 70% (Moderate) irrigation level. Previous studies have also found a reduction in needle area and/or length under drought conditions (Fischer and Turner, 1978; Pinto et al., 2012). The reason the 70% level resulted in greater needle area may be that the 55% irrigation level was not extreme enough or that the 85% level was excessively irrigated. Regardless, a target characteristic for outplanting on dry, harsh sites may be smaller needle area because it will likely have less transpirational demand. However, this also depends on stomatal density, for which lower density would typically result in decreased levels of transpiration and better adaptation to drought environments (Hepworth et al., 2015). Results from our study showed that only the Philadelphia Canyon source had a significant reduction in stomatal density with decreasing irrigation. The Valle Vidal and Manzano Canyon sources showed a trend of decreasing stomatal density with decreasing irrigation level. This suggests that seedlings treated with the 55% (Low) irrigation level may be better adapted to dry outplanting sites.

In contrast to most of the morphological characteristics, seedling physiological characteristics (i.e., photosynthetic rate and water use efficiency) showed positive responses under both the 55% and 70% irrigation levels. Two studies observed a higher production rate of photosynthetic pigments reported for seedlings under greater moisture stress (Vilagrosa et al., 2010; Taïbi et al., 2017). While a higher photosynthetic rate or capacity may translate to advantages in terms of CO_2_ assimilation and overall plant growth, we did not observe this in our study. Due to the cyclic nature of irrigation application, we suspect opportunistic photosynthetic responses to the favorable conditions of when media moisture was high. These instances were likely short in duration as indicated by water use efficiency and the long-term, integrative carbon isotope data that indicate greater duration of stomatal closure. In the field, Pinto et al. (2016) found that ponderosa pine seedlings responded with almost a four-fold increase of photosynthetic rates with the return of soil moisture following drought. Other studies have also observed increased water use efficiency under drought conditions, and this was both associated with reduced stomatal conductance and stomatal density (DeLucia and Heckathorn, 1989; Pinto et al., 2012; Hepworth et al., 2015).

Currently, there are no known studies measuring hydraulic activity of xylem tracheids in *P. ponderosa* by using staining methods (Jacobsen et al., 2015). Unfortunately, results from the NCE suggested the crystal violet dye solution was not an effective dye to stain the active xylem structure due to the extremely dark coloration and lateral bleeding of the dye. This limited the ability of this study to assess the percentage of hydraulically active xylem with a high degree of precision. However, the staining method still proved effective for measuring xylem flow velocity. Results from this study showed an increase in xylem flow velocity with decreasing irrigation level. This observation might have contributed to increased tracheid formation, although measurements on tracheid characteristics were not taken in our study. Taïbi et al. (2017) found that xylem flow rates in *Pinus halepensis* were greater with tracheids that were shorter and had smaller diameters. However, the results from the NCE contrast with those of other studies that found decreases in xylem flow rates to correspond with increases in plant moisture stress (Sperry and Tyree, 1988; Lovisolo and Schubert, 1998).

### Simulated outplanting experiment

One of the more critical results from the SOE, similar to the NCE, was the continued lack of interaction effects between seed source and irrigation treatment for most response variables. This suggests that irrigation nursery practices remain effective across this range of seed sources beyond the nursery phase and into the outplanting phase. Given that few interactions were observed between seed source and irrigation level, discussions here will mainly focus on irrigation and simulated drought treatments.

At the initial harvest for the SOE, the Low and Moderate irrigation levels significantly increased seedling morphological responses such as height, diameter, plant biomass, and needle area. This contrasts with the findings of Royo et al. (2001); Olivas-García et al. (2000), and Guarnaschelli et al. (2006), where drought conditions resulted in decreased morphological responses. However, the positive physiological responses seen in the NCE may have translated to the positive morphological responses observed in the SOE. Increases in photosynthetic rate and capacity as well as decreases in total needle area are all seedling characteristics capable of improving outplanting performance on dry sites (Pinto et al., 2012; Hepworth et al., 2015). Given that there was no interaction between the nursery irrigation treatments and the simulated drought treatments for all morphological responses, those seedlings exposed to the lower irrigation levels (Low and Moderate) performed better under both drought conditions and optimal soil moisture.

As with morphological responses, plant vascular function was significantly altered under the lower irrigation treatment levels. Xylem established during the nursery phase (Year-1) showed that the Moderate irrigation level resulted in the greatest percentage of active xylem. For xylem established during the outplanting phase (Year-2), both the Low and Moderate irrigation levels had greater areas of active xylem compared to the high irrigation level. By increasing the total area of active xylem, as seen in both Year-1 and Year-2 xylem under the low irrigation treatment level, the proportion of active xylem that may be potentially lost during any given cavitation event (i.e., drought period) decreases (Hacke et al., 2006; Venturas et al., 2017). This alteration of xylem hydraulic properties may offer targeted seedling characteristics for restoration of dry, harsh outplanting sites.

## Conclusion

During the NCE, *P. ponderosa* seedlings exposed to limited irrigation were smaller for many morphological effects (e.g., height, RCD, FOLR, root:shoot, and needle area). Some physiological responses, however, improved under the lower irrigation levels. These positive effects in terms of increased photosynthetic rate and water use efficiency coupled with decreased needle area may be target plant characteristics needed to improve outplanting success as was observed in the SOE. This experiment was the first to test conifer drought conditioning for the entire first growing season in the nursery. However, there are some suggestions that can be made for future research directions. The first relates to the dye solution used in the xylem staining method. It will be important to find the right dye material and associated staining procedures that will improve visual assessment of active xylem structures and tracheid diameters in this species. The second opportunity is to measure plant moisture stress levels both during the nursery phase and in outplanted seedlings. This can be done by using pressure chambers to measure xylem water potential at various times during the growth period and/or by carbon isotope analysis as a more integrated measure of plant stress. Future studies in the area of drought conditioning should address these opportunities as well as focus on a larger genetic component by including enough seed sources to meet the requirements of typical provenance tests.

## Data availability statement

The raw data supporting the conclusions of this article will be made available by the authors, without undue reservation.

## Author contributions

JP, JS, and OB all contributed equally to the design, implementation, analysis, and writing of this research. GE contributed to implementation, analysis, and writing. All authors contributed to the article and approved the submitted version.
